# Comparative assessment of probiotics and monensin in the prophylaxis of acute ruminal lactic acidosis in sheep

**DOI:** 10.1186/s12917-017-1264-4

**Published:** 2018-01-09

**Authors:** Leonardo Frasson Reis, Rejane Santos Sousa, Francisco Leonardo Costa Oliveira, Frederico Augusto Mazzocca Lopes Rodrigues, Carolina Akiko Sato Cabral Araújo, Enoch Brandão Souza Meira-Júnior, Raimundo Alves Barrêto-Júnior, Clara Satsuki Mori, Antonio Humberto Hamad Minervino, Enrico Lippi Ortolani

**Affiliations:** 1 Department of Clinical Science, Faculty of Veterinary Medicine and Animal Science, University of São Paulo, Av. Prof. Orlando Marques de Paiva, 87, Cidade Universitária, CEP, São Paulo, SP 05508-270 Brazil; 2 Department of Animal Science, Federal Rural University of the Semiarid Region, Av. Francisco Mota, s/n° - Bairro Pres. Costa e Silva, CEP, Mossoró, RN 59625-900 Brazil; 3 Laboratório de Sanidade Animal, Universidade Federal do Oeste do Pará (LARSANA/UFOPA), Av. Vera Paz S/N, Salé, CEP, Santarém, PA 68000-000 Brazil

**Keywords:** Prevention, Additives, Ionophores, Ruminal pH

## Abstract

**Background:**

Acute ruminal lactic acidosis (ARLA) is a major nutritional and metabolic disorder usually characterized by excessive or non-adapted intake of diets rich in nonstructural carbohydrates. Feed additives that regulate the ruminal environment have been used to prevent ARLA, such as ionophores and, more recently, yeast culture. Thus, we aimed to compare the efficacy of a yeast-based culture (*Saccharomyces cerevisiae*) with that of monensin sodium in the prevention of ARLA in sheep. Eighteen male, crossbred, rumen-cannulated sheep were randomly distributed into three groups of six animals: control, yeast culture and monensin. Thirty days after the start of supplementation with yeast culture (4 × 10^9^ cfu/animal/day of *S. cerevisiae*) and monensin (33 mg/kg of total dry matter intake), 15 g/kg BW of sucrose was administered directly into the rumen of the animals to induce ARLA. Samples of blood and ruminal fluid were collected at the following time points: at baseline (T0 h) immediately before the induction of ARLA; 6 h (T6 h); 12 h (T12 h); 18 h (T18 h); 24 h (T24 h); 36 h (T36 h); and 48 h (T48 h) after ARLA induction.

**Results:**

Ruminal pH was higher in monensin group at T12 h and in yeast culture group at T36 h when compared to control group. Lower values of L-Lactate were found at yeast culture group at T24 h and T36 h. Monensin showed prophylactic effect by decreasing the rate of ruminal pH decline and occasionally reducing ruminal acidosis, whereas probiotics resulted in less accumulation of lactic acid in the rumen and a lower degree of systemic acidosis.

**Conclusion:**

The use of yeast culture can be beneficial in the prevention and treatment of ARLA in sheep, because it can effectively reduce the accumulation of lactic acid, and thereby increase ruminal pH and reduce ruminal osmolarity. On the other hand, monensin showed prophylactic effect by decreasing the rate of ruminal pH decline and occasionally reducing ruminal acidosis, however, it did not directly prevent these conditions.

## Background

Sheep use complex carbohydrates (structural and nonstructural) as their main source of energy, and require these nutrients in the diet to maintain healthy, stable ruminal conditions [1]. Nevertheless, feeding practices that use nonstructural carbohydrates aimed at achieving high levels of productivity, have led to increased levels of fermentation and the production of organic acids, and a subsequent reduction of ruminal pH [2]. Although increased acid production can sometimes be desirable, this type of nutritional management can challenge the equilibrium of the ruminal ecosystem and compromise animal health [3].

Acute ruminal lactic acidosis (ARLA) is a major nutritional and metabolic disorder usually characterized by excessive or non-adapted intake of diets rich in nonstructural carbohydrate. The excessive ingestion of nonstructural carbohydrates, rich in sugar, starch and pectin, causes sudden, exponential growth of specific Gram-positive bacteria (*Streptococcus bovis* and *Lactobacillus* sp) that generate intense production of lactic acid and cause a sharp drop in ruminal pH, which then interferes with the activity and survival of lactate-utilizing (Gram-negative) bacteria that transform lactic acid into inactive substances [4, 5].

It is therefore necessary to use tools that maximize the potential for production, by facilitating small adjustments to the process of ruminal fermentation, while using diets rich in nonstructural carbohydrates [6]. In this context, feed additives have been used to prevent ARLA, such as ionophores and yeast culture that regulate the ruminal environment to increase feed efficiency [7, 8].

Ionophores are antimicrobial compounds produced by various strains of *Streptomyces* sp. that have a selective inhibitory action on Gram-positive bacteria and allow the survival of Gram-negative bacteria. Monensin sodium is the most commonly used ionophore in the prevention of ARLA in beef cattle [9]. Nevertheless, monensin is not approved for use in ruminants in all countries [10, 11].

Yeast culture are additives composed of live microorganisms that improve animal productivity and promote the growth of bacteria (particularly cellulolytic and lactate-utilizing bacteria) and protozoa in the rumen [4], thus, it could be used in substitution of ionophores in ruminants.

Although *Saccharomyces cerevisiae* has been tested in several in vitro and in vivo trials to increase the activity of lactate-utilizing bacteria [12–16], to our knowledge, no study has been conducted to assess the preventive effects of probiotics in ARLA under controlled experimental conditions, particularly in sheep. Moreover, to our knowledge, there are no studies that compare the action of these yeast culture with that of monensin sodium in the prophylaxis and treatment of ARLA in sheep.

Therefore, the present study aimed to compare the effects of a yeast culture that contains *Saccharomyces cerevisiae* with those of monensin sodium on ruminal and hematological variables and assess the efficacy of both additives in the prevention and treatment of sheep affected by ARLA.

## Methods

The sheep used in the experiment were purchased at properties near the city of São Paulo, obeying the norms of acquisition of animals for experimentation and with the aproval of the Ethics Committee on Animal Research of the Faculty of Veterinary Medicine and Animal Science, University of São Paulo (Protocol no. 1587/2009).

### Animals and diet

Eighteen male 24-month-old, Santa Inês crossbred sheep with a mean body weight of 45 ± 1.2 kg were used. Sixty days before the start of the study, all animals were treated with a moxidectin-based endectocide (Cydectin, Zoetis Animal Health, São Paulo, Brazil) and were surgically fitted with a ruminal silicone cannula. The animals were subsequently allowed to recover and adapt to the feeding protocol.

During the adaptation period and throughout the study, the sheep were fed a basal diet calculated at 2.7% body weight, dry matter (DM) basis, with 75% being coast-cross grass (*Cynodon dactylon*) and 25% commercial concentrate with 14% crude protein (Fri-Sheep 22/70, Nutreco Nutrição Animal, Pitangueiras, Brazil). The hay and concentrate were mixed and the diet was offered at once in the morning. The animals had free access to mineral salt (Ovinofós, Tortuga, São Paulo, Brazil) and water. The sheep were weighed every week and the diet was correct accordingly.

### Study design

The experimental design was completely randomized with distribution of the 18 sheep into three groups of six animals: the control, yeast culture, and monensin groups. The control group received the basal diet; the yeast culture group received the basal diet and 5 g of yeast culture/animal/day (Yea-Sacc^®^ 1026, Alltech SA, Araucária, Brazil) that resulted in a total inoculation of 4 × 10^9^ cfu/animal/day of *Saccharomyces cerevisiae* strain 1026; and the monensin group received 33 mg of monensin sodium (Rumensin^®^, Elanco, São Paulo, Brazil) per kg of diet [17], i.e. a sheep weighing 45 kg BW received daily 1.215 kg of diet (DM) and 40.1 mg of sodium monensin. Yeast culture and monensin were both administered directly into the rumen via the ruminal cannula immediately after the animals were fed. Monensin dose were adjusted weekly according the diet.

Ruminal lactic acidosis was experimentally induced, 30 days after the additives were introduced. The experimental model entailed the administration of sucrose into the rumen [18], according to the modifications recommended by Afonso et al. [17] for sheep. For this purpose, 15 g of sucrose per kg of body weight was administered directly into the rumen.

Blood and ruminal fluid samples were collected at the following time points: at baseline (T0 h) immediately before the induction of ARLA, and at 6 (T6 h), 12 (T12 h), 18 (T18 h), 24 (T24 h), 36 (T36 h) and 48 h (T48 h) after induction of ARLA. Starting at T24 h until T48 h, fresh grass was offered to each animal to evaluate the presence or absence of appetite.

To protect the welfare of the animals, all sheep that exhibited clinical signs of systemic acidosis and had blood pH ≤ 7.2 were treated at T24 h. Treatment consist of intravenous infusion of isotonic saline solution (20 mL/kg BW) and isotonic sodium bicarbonate (1.3%), according to the classic formula of buffer replacement [19]. In addition, at T24 h, the control group received 5 g of sodium chloride into the rumen, and the monensin and yeast culture groups received the respective additives at the aforementioned doses.

### Ruminal content samples

Aliquots of ruminal contents were collected at the specified time points using a probe placed in the ventral sac of the rumen and a vacuum pump. Approximately 100 mL of fluid was collected, filtered with gauze, and frozen at −20 °C for subsequent determination of osmolarity and concentration of L-lactate.

### Ruminal evaluation by the continuous telemetry system

During the induction of ARLA, rumen pH and temperature were measured continuously by a telemetric system of data acquisition using a device composed of a submersible electrode (model PHE-6510), a data logger (model OM-CP-PH10), an interface cable (model OM-CP-IFC110) and the Omega 2.04.6 software (Omega Engineering Inc., Campinas, Brazil), according to the recommendations of Alzahal et al. [20]. The electrode was placed in the animals at T0 h and remained within the ventral sac of the rumen for 48 consecutive hours. Ruminal temperature and pH were recorded every 5 min (with sensitivities of 0.01 °C and 0.01, respectively).

### Blood samples

Arterial blood samples were collected from auricular artery at all time points using a scalp (23G) coupled to heparinized syringes. Blood gas was performed in an automated analyzer (Cobas 212, Roche Diagnostics, Basel, Switzerland). The following parameters were evaluated: blood pH, concentration of bicarbonate, and base excess (BE). The results were corrected according to the rectal temperature of the animal [21, 22].

Plasma samples were collected in tubes containing sodium fluoride to determine L-lactate and glucose concentrations, whereas serum samples were used to determine serum osmolarity.

### Laboratory analysis

The levels of plasma and ruminal L-lactate and plasma glucose were measured using commercial kits (Randox^®^, Antrium, UK) in an automated biochemistry analyzer (RX Daytona, Randox^®^, Antrium, UK). Determination of serum and ruminal osmolarities were based on the freezing point method and conducted in an osmometer (Advanced Micro-Osmometer 3300, Advanced™ Instruments, Norwood, USA).

The methylene blue reduction test was performed in the ruminal fluid at T36 h and T48 h using the classic technique described by Dirksen et al. [23].

### Statistical analysis

Statistical analysis was performed using the SAS 9.3 statistical software. The tests of normality of residuals and homogeneity of variance were performed, the variables that met the assumptions were subjected to analysis of variance using the PROC MIXED procedure for measurements repeated over time. For each variable, the effect of treatment, time and interaction between treatment and time was analysed. It was considered the Akaike Information Criterion (AIC) for choosing the best covariance structure. In the evaluation of rumen osmolarity, T0 h values were used as covariate since the presented statistical difference between groups. Comparisons between the means of groups at each interval (P) were performed using the least square means (LS Means) test. Correlation coefficients were calculated and regression equations were derived to determine the relationship between two variables. The level of significance was set at 5%.

## Results

Values of pH, temperature, osmolarity and concentration of L-lactic in the ruminal content recorded throughout the study are presented in Table 1. Animals of the probiotic group had higher ruminal pH values (Trat = 0.01; Time = 0.01; Trat*Time = 0.14) when compared to the control and monensin groups. At T12 h, the pH of ruminal contents in the monensin group was higher than that in the control group, but identical to that in the yeast culture group. On the other hand, at T36 h and T48 h, the pH of ruminal contents in the yeast culture group was significantly higher than that in the control group and identical to that in the monensin group. There were significant differences in intraruminal temperature among the groups at three time points, whereas the animals of the monensin and probiotic groups presented higher values (Trat = 0.03, Time = 0.01, Trat*Time = 0.13). At T12 h, the ruminal temperature of the control group was higher than that of the other groups, whereas at T36 h and T48 h, the ruminal temperature of the yeast culture and monensin groups was higher than that of the control group.

**Table 1:** Means and standard deviations of ruminal variables in the control group and in the groups supplemented with monensin and yeast culture at various time points

		Time points								p	
Variable	Groups	T0 h	T6 h	T12 h	T18 h	T24 h	T36 h	T48 h	Trat	Time	Trat*Time
pH	Control	6.60 ± 0.12^a^	4.81 ± 0.29^c^	4.45 ± 0.05^Bcd^	4.50 ± 0.30^cd^	4.35 ± 0.30^d^	4.76 ± 0.57^Bc^	5.70 ± 0.75^Bc^	0.01	0.01	0.14
	Monensin	6.79 ± 0.23^a^	4.80 ± 0.13^c^	5.21 ± 0.53^Ac^	4.96 ± 0.55^c^	4.79 ± 0.37^c^	5.47 ± 0.39^ABbc^	6.41 ± 0.61^ABab^			
	Yeast	6.72 ± 0.17^a^	4.87 ± 0.18^b^	4.80 ± 0.12^ABb^	4.50 ± 0.35^b^	4.85 ± 0.77^b^	6.36 ± 0.59^Aa^	7.08 ± 0.35^Aa^			
Temperature (°C)	Control	38.8 ± 0.6^c^	40.4 ± 0.5^b^	41.1 ± 0.2^Aa^	38.4 ± 0.7^cd^	38.1 ± 0.6^cd^	37.8 ± 1.5^Bde^	37.7 ± 1.5^Be^	0.03	0.01	0.13
	Monensin	38.5 ± 0.6^b^	40.0 ± 0.5^a^	40.4 ± 0.2^Ba^	39.7 ± 0.7^ab^	39.5 ± 0.6^ab^	40.2 ± 1.5^Aa^	40.0 ± 1.5^Aa^			
	Yeast	39.0 ± 0.2^b^	40.7 ± 0.6^a^	40.6 ± 0.4^ABa^	39.7 ± 1.1^ab^	39.5 ± 1.6^ab^	39.9 ± 0.6^Aab^	40.0 ± 0.4^Aab^			
Osmolarity* (mOsm/L)	Control	274 ± 19^b^	453 ± 75^Aa^	357 ± 92^ab^	309 ± 29^A^	328 ± 67^Ab^	306 ± 14^Ab^	309 ± 21^Aab^	0.02	0.01	0.09
	Monensin	253 ± 12	377 ± 89^B^	284 ± 75	307 ± 81^A^	339 ± 42^A^	256 ± 42^AB^	218 ± 34^B^			
	Yeast	206 ± 13^b^	293 ± 42^Bab^	299 ± 42^a^	278 ± 77^Bab^	234 ± 26^Bab^	205 ± 53^Bab^	202 ± 22^Bab^			
Lactate L (mmol/L)	Control	0.47 ± 0.1^d^	33.6 ± 11^bcd^	68.3 ± 27^ab^	69.4 ± 30^ab^	81.2 ± 33^Aa^	47.8 ± 20^Aabc^	15.2 ± 42^Acd^	0.01	0.01	0.14
	Monensin	0.34 ± 0.1^c^	30.3 ± 12^ab^	39.9 ± 17^ab^	42.9 ± 18^a^	41.6 ± 12^ABa^	26.2 ± 18^Aab^	16.2 ± 80^Ac^			
	Yeast	0.26 ± 0.3^c^	22.3 ± 52^bc^	38.9 ± 11^ab^	51.0 ± 19^a^	33.0 ± 20^Bab^	1.39 ± 0.9^Bc^	0.45 ± 0.1^Bc^			

Upper-case letters in columns denote statistically significant differences (*p* < 0.05) between groups throughout least square means test. Lower-case letters in rows denote statistically significant differences (*p* < 0.05) between time points by analysis of variance for measurements repeated over time. * Osmolarity presented difference in the T0 h, thus those values were included as covariates in the statistical analyses of this variable

At baseline (T0 h) the probiotic group presented lower values of ruminal osmolarity and this time point became a covariate in the analyses. The highest values of ruminal osmolarity were observed at T6 h among all groups and at this time point, the control group exhibited higher values of ruminal osmolarity than the other groups (Trat = 0.02; Time = 0.01; Trat*Time = 0.09). At the time points T18 h and T24 h the osmolarity in those animals that received yeast culture supplementation was lower than that in the control and monensin groups; and there was no significant difference between the two latter groups. However, at T48 h, osmolarity in the monensin and yeast culture groups was lower than that in the control group.

Lower concentrations of rumen lactate were detected in the animals of the probiotic group when compared to the monensin and control groups (Trat = 0.01; Time = 0.01; Trat*Time = 0.14). From T6 h onwards, there was an increase in the concentration of L-lactic acid in the ruminal contents of all groups, with maximum values being observed at T18 h in the yeast culture and monensin groups, and at T24 h in the control group. Between T24 h and T48 h, L-lactate levels were higher in the control group than in the yeast culture group. At T48 h, the mean concentration of L-lactate in the ruminal contents of the yeast culture group was similar to that of the baseline value, an observation that was not evident in the other groups.

The results of the methylene blue reduction test were lower (*p* = 0.040) in the yeast culture group at T36 h and T48 h (5 min and <3 min, respectively) than those in the monensin group (30 min and 10 min, respectively), and in the control group (40 min and 15 min, respectively); however, these differences were not statistically significant.

**Fig. 1:**
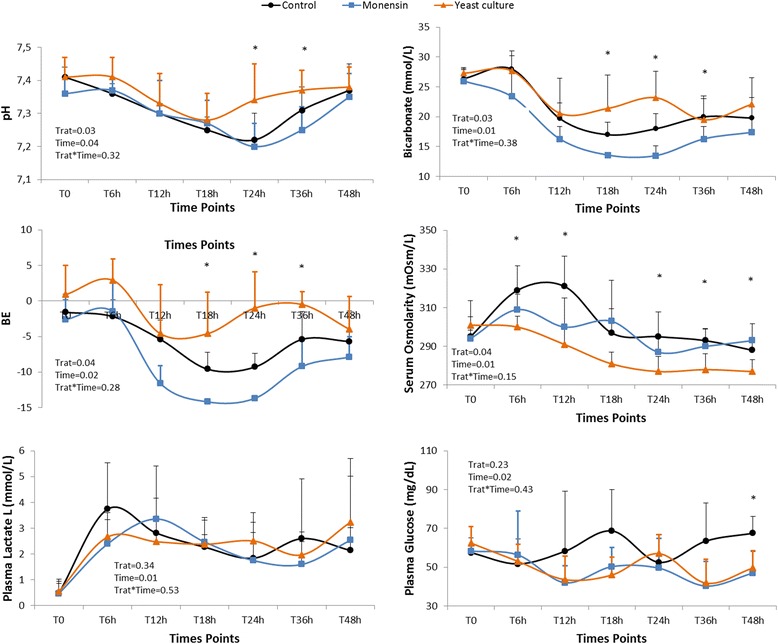
Means and standard deviation of blood variables of the control group and the groups supplemented with monensin or yeast after induction of acute ruminal lactic acidosis. Blood pH (Trat = 0.03; Time = 0.04; Trat*Time = 0.32); bicarbonate ((Trat = 0.03; Time = 0.01; Trat*Time = 0.38); base excess (BE) (Trat = 0.04; Time = 0.02; Trat*Time = 0.28); serum osmolarity (Trat = 0.04; Time = 0.01; Trat*Time = 0.15); Plasma lactate (Trat = 0.34; Time = 0.01; Trat*Time = 0.53) and plasma glucose (Trat = 0.23; Time = 0.02; Trat*Time = 0.43). *Denotes statistically significant difference (*p* < 0.05) between the experimental groups at the time of evaluation throughout least square means test

Figure 1 shows the results of the analyzed blood variables. From T12 h onwards, all groups had blood pH values below baseline values (T0 h). The values of blood pH from the yeast group were higher than control and monensin groups (Trat = 0.03; Time = 0.04; Trat*Time = 0.32). The base excess was lower in the yeast culture group (Trat = 0.04; Time = 0.02; Trat*Time = 0.28) than other groups, whereas the concentration of bicarbonate was lower in the control and monensin groups than it was in the yeast culture group (Trat = 0.03; Time = 0.01; Trat*Time = 0.38).

There were not significant differences in plasma L-lactate concentration among the groups (Trat = 0.34; Time = 0.01; Trat*Time = 0.53). The comparison between time points showed that there were significant differences only within the control group, namely a significant increase at T6 h (the time point at which the concentration of L-lactate reached its maximum in the control group).

Serum osmolarity in the control group was higher than that in the yeast culture group (Trat = 0.04; Time = 0.01; Trat*Time = 0.15). At T6 h and T12 h yeast culture group exhibiting the lowest osmolarity values of the three groups. In addition, serum osmolarity of the yeast culture group was significantly different from that of the control group, but showed no significant difference from that of the monensin group. At T48 h, the concentration of plasma glucose was highest in the control group and there were no significant differences between the monensin and yeast culture groups, but no treatment effect was detected for this variable among the studied groups (Trat = 0.23; Time = 0.02; Trat*Time = 0.43).

Three animals from the control group and two from the monensin group required treatment at T24 h. This animals exhibited absence of appetite at T24 h. Only one animal (from control group) had severe acidosis, with a blood pH of 7.07. At T36 h, all treated animals had increased blood pH. No animals in the probiotic group required support treatment.

## Discussion

The methods of the present study effectively induced ARLA, achieving significant ruminal pH values, specifically a mean of 4.0 at T24 h in the control group, which indicates severe ruminal acidosis [19]. The results observed in the control group suggest that the amount of substrate used facilitated continuous fermentation of sucrose up to T24 h, with decrease in ruminal pH and accumulation of lactic acid in the rumen.

The results of ruminal pH showed that monensin had a positive effect at T12 h by preventing a sharp decline in pH. This possibly occurs due a reduced production of L-lactate and short-chain fatty acid (SCFA), since monensin modulates the activity of lactic acid bacteria that produce SCFAs [9]. At T12 h, the yeast culture had an intermediate effect, probably stimulating the activity of lactic acid consuming bacteria, but exerting less pronounced effects on SCFA-producing bacteria [24].

However, at T24 h, when the concentrations of acids reached maximum values, the yeast culture indirectly promoted a trend of increasing ruminal pH through a significant reduction in the production of L-lactic acid. This effect was probably due to competition between the *Saccharomyces cerevisiae* yeast and the fermenting bacteria that produce lactic acid (particularly *Lactobacillus sp.*) [25], or competition between the *Saccharomyces cerevisiae* yeast and the substrates made available by the yeast for the growth of those bacteria [24]. The yeast culture exerted its greatest effects between T24 h and T48 h, during recovery from ruminal acidosis. During this period, reductions in the concentration of L-lactic acid in the rumen were significant in the yeast culture group. According to Callaway and Martin [25], probiotic yeasts have a symbiotic relationship with lactic acid consuming bacteria (*Selenomonas ruminantium*, *Megasphaera elsdenii*); suggesting that yeasts provide B-complex vitamins and amino acids that stimulate the growth of these bacteria, which utilize the ruminal lactate for metabolism. This effect seemed to be greater after T18 h, when the mean ruminal pH reached a value of 4.5, which is the ideal pH for the multiplication of yeasts [25]. The reduction in the concentration of L-lactic acid and the increase in ruminal pH suggests that the use of yeast culture could be used as ancillary treatment in sheep with ARLA.

Variations in ruminal temperature have been considered only recently, mainly because temperature data is difficult to obtain. According to Alzahal et al. [26], ruminal temperature can be useful in the diagnosis of ruminal acidosis by volatile fatty acids (subacute acidosis). They assert that under these ruminal conditions (pH between 5.6 and 5.0), ruminal temperature increases beyond 39.2 °C, reaching up to 41 °C, and correlates negatively with pH (*r* = −0.87). In the present study, ruminal temperature caused by ARLA was measured, at pH < 5.0, and a quadratic relationship between pH and temperature (*r* = 0.71) was observed. At the peak of fermentation (T18 h), when the pH was approximately 4.2, ruminal temperature was also at its maximum (40.5 °C), which suggesting an intrinsic relationship between these two variables. At T12 h, the mean temperature in the group supplemented with monensin was lower than that in the control group, which supports the hypothesis that this additive reduces ruminal fermentation at this time point.

However, at T24 h, when ruminal pH was still very low, there was a drop in ruminal temperature. This probably occurred because the infused substrate, sucrose, had already been completely consumed, reducing ruminal activity, especially in the control group. The mean ruminal temperatures at T36 h and T48 h in the control group were lower than those in the monensin and probiotic groups, which suggest that there was a biochemical disruption of ruminal flora in the control group.

At T36 h and T48 h, the reaction time of the methylene blue reduction test on rumen fluid was shorter in the yeast culture group than in the other groups. In addition, no animal in the yeast culture group exhibited absence of appetite, whereas 50% of the animals in the control group and 33% in the monensin group exhibited this condition. One possible hypothesis to explain those finding is that the consumption of oxygen by *S. cerevisiae* favors a return to the normal status (anaerobiosis in particular) of the ruminal environment [27].

As expected, the concentration of ruminal L-lactate significantly affected ruminal osmolarity (*r* = 0.72). Osmolarity in the yeast culture group was lower than that in the control group at T0 h, T24 h, T36 h, and T48 h; the low baseline values observed in the group supplemented with yeast culture are noteworthy. The reason for this difference is unknown and further studies are necessary to interpret these results.

ARLA caused mild to moderate systemic acidosis in most animals, with the exception of one sheep in the control group whose blood pH at T24 h was 7.07 (acute systemic acidosis). Treatment with sodium bicarbonate buffer partially corrected metabolic acidosis in the control and monensin groups at T36 h, because a residual amount of ruminal lactic acid prevented full adjustment of the systemic pH. Moreover, the levels of bicarbonate and base excess in the animals that received yeast culture were higher between T24 h and T36 h, which coincided with the lowest levels of ruminal L-lactic acid in this group.

A trend of increasing concentration of plasma L-lactate over time was observed in the control group. The plasma lactate probably originated from the rumen, because the coefficient of determination between these variables was 0.77. Ruminal L-lactate may led to an increase in the concentration of plasma glucose, especially in the control group, as evidenced by a quadratic relationship (*r*^2^ = 0.54) between these two variables.

## Conclusion

The use of yeast culture can be beneficial in the prevention and treatment of ARLA in sheep, because it can effectively reduce the accumulation of lactic acid, and thereby increase ruminal pH and reduce ruminal osmolarity. On the other hand, monensin showed prophylactic effect by decreasing the rate of ruminal pH decline and occasionally reducing ruminal acidosis, however, it did not directly prevent these conditions.

## Abbreviations

ALRA: ruminal lactic acidosis
SCFA: short-chain fatty acid
